# Energy Storage, Power Management, and Applications of Triboelectric Nanogenerators for Self-Powered Systems: A Review

**DOI:** 10.3390/mi16101170

**Published:** 2025-10-15

**Authors:** Xiong Dien, Nurulazlina Ramli, Tzer Hwai Gilbert Thio, Zhuanqing Yang, Siyu Hu, Xiang He

**Affiliations:** 1 School of Information Engineering, Chongqing Vocational and Technical University of Mechatronics, Chongqing 402760, China; sukd2301023@segi4u.my (X.D.); husiyu0222@gmail.com (S.H.); 2 Centre for Sustainability in Advanced Electrical and Electronics Systems (CSAEES), Faculty of Engineering and the Built Environment, SEGi University, Kota Damansara, Petaling Jaya 47810, Selangor, Malaysia; azlinaramli@segi.edu.my (N.R.); gilbertthioth@segi.edu.my (T.H.G.T.); 3 School of Big Data and Internet of Things, Chongqing Vocational Institute of Engineering, Chongqing 402260, China; yangzq@cqvie.edu.cn

**Keywords:** TENG, energy storage, power management, applications

## Abstract

Triboelectric nanogenerators (TENGs) have emerged as efficient mechanical-energy harvesters with advantages—simple architectures, broad material compatibility, low cost, and strong environmental tolerance—positioning them as key enablers of self-powered systems. This review synthesizes recent progress in energy-storage interfaces, power management, and system-level integration for TENGs. We analyze how intrinsic source characteristics—high output voltage, low current, large internal impedance, and pulsed waveforms—complicate efficient charge extraction and utilization. Accordingly, this work highlights a variety of power-conditioning approaches, including advanced rectification, multistage buffering, impedance transformation/matching, and voltage regulation. Moreover, recent developments in the integration of TENGs with storage elements, cover hybrid topologies and flexible architectures. Application case studies in wearable electronics, environmental monitoring, smart-home security, and human–machine interfaces illustrate the dual roles of TENGs as power sources and self-driven sensors. Finally, we outline research priorities: miniaturized and integrated power-management circuits, AI-assisted adaptive control, multimodal hybrid storage platforms, load-adaptive power delivery, and flexible, biocompatible encapsulation. Overall, this review provides a consolidated view of state-of-the-art TENG-based self-powered systems and practical guidance toward real-world deployment.

## 1. Introduction

As an emerging energy-harvesting technology based on the coupled effect of contact electrification and electrostatic induction, TENGs exhibit distinctive technological positioning and multi-scenario adaptability within self-powered systems [1,2]. Compared with conventional electromagnetic generators, piezoelectric materials, and other harvesting approaches, TENGs have significant advantages in terms of power density, material cost, and flexible integration. Under low-frequency mechanical excitation, their power density can reach up to 500 W/m^2^, far exceeding that of electromagnetic generators [3]. Polymer–metal composite friction layers enable low-cost fabrication, reducing the cost per unit of harvested energy by 60% compared with piezoelectric ceramics [4]. In addition, TENGs enable flexible substrate integration, and micro- and nanostructure engineering allows them to achieve bending radii as small as 0.01 mm, thereby satisfying the deformation requirements of wearable electronics [5,6,7].

**Figure 1:**
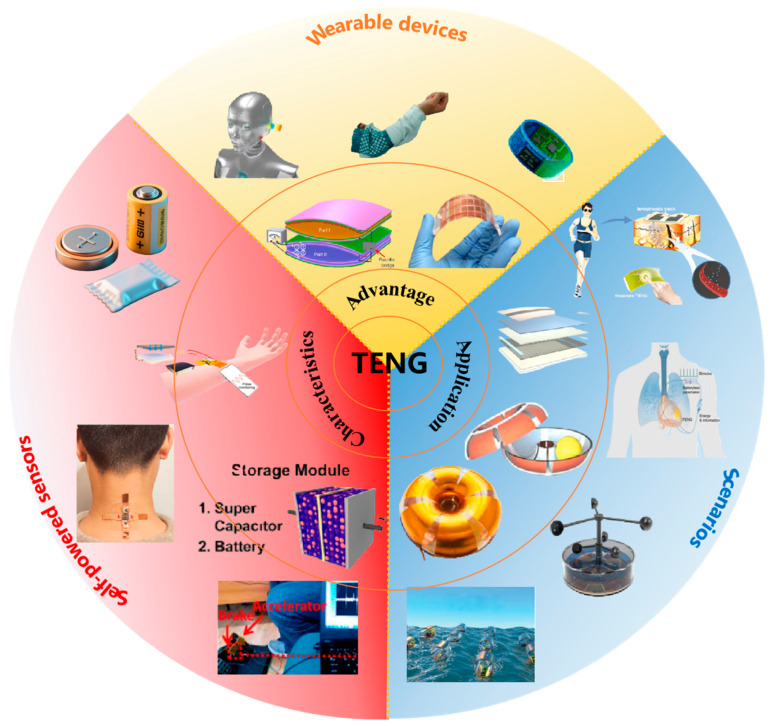
Schematic illustration of the multifunctional roles of TENGs as both energy harvesters and self-driven sensors in wearable, environmental, and biomedical systems. Reproduced with permission from ref. [12]. Copyright 2019, Elsevier. Reproduced with permission from ref. [13]. Copyright 2021, John Wiley and Sons. Reproduced with permission from ref. [14]. Copyright 2020, John Wiley and Sons. Reproduced with permission from ref. [15]. Copyright 2021, Elsevier. Reproduced with permission from ref. [16]. Copyright 2021, American Chemical Society. Reproduced with permission from ref. [17]. Copyright 2018, Elsevier.

TENG’s dual roles as energy harvester and self-driven sensor enable an “integrated sensing–energy supply” paradigm for IoT and distributed networks [4]. Unlike short-lived, costly, and polluting batteries, TENG-based self-powered sensors facilitate large-scale deployment while reducing maintenance requirements in smart industrial IoT [8]. By directly converting ambient mechanical energy into electrical energy, TENGs produce output signals that are linearly correlated with mechanical stimulus parameters, thereby realizing the synergistic optimization of “energy generation–signal sensing” [9]. For example, in wearable healthcare applications, TENGs capture energy from gait and simultaneously enable self-driven monitoring of heart rate signals, effectively addressing the system-complexity challenges associated with the “power-sensing” separation in conventional sensors, as shown in Figure 1 [10]. Lu reported on an ocean-buoy system in which a magnetically levitated TENG exhibited < 8% output fluctuation across −40 to 85 °C and achieved continuous, maintenance-free monitoring for 12 months [11].

The high open-circuit voltage and low short-circuit current characteristics of TENGs are inherently mismatched with the low-voltage, high-current requirements of electronic devices, leading to energy-transfer efficiencies typically below 30% [18]. In addition, the irregularities in pulsed output and mechanical excitation impose stringent challenges on efficient energy-storage technologies and dynamic power-management circuits (PMCs) [19].

Despite the many advantages of TENGs in energy harvesting and self-driven sensing, their output impedance is typically as high as 10–100 MΩ, with output voltages reaching the kilovolt level while currents remain in the microampere range [20]. High-efficiency energy-storage circuits can increase conversion efficiency from below 10% to 42.5% and reduce output impedance by 3–5 orders of magnitude [21]. However, TENGs cannot directly and stably drive conventional electronics or sensors. When driving conventional electronics directly, more than 70% of harvested energy can be dissipated as Joule heat, and irregular mechanical excitation further exacerbates the power supply instability [22].

To fully utilize the energy generated by TENGs and provide a stable and reliable power supply for the subsequent circuits, the design of the energy-storage circuit is crucial. Efficient energy-storage circuits not only accumulate the intermittent output of TENGs and deliver it at stable voltage and current levels when needed but also filter and regulate signals to improve their quality and stability [23]. Therefore, energy storage plays a central role in bridging “mechanical energy–electrical energy–sensing signal.” By rationally designing storage architectures, the limitations of TENG output can be mitigated, thereby maximizing their potential in self-powered sensors [24].

The selection and parameter optimization of storage components directly determine the performance of energy-storage circuits. Commonly used elements include capacitors, supercapacitors, and rechargeable batteries, each suited for different scenarios. Capacitors feature ultrafast charging and discharging rates and long cycle life, but their low energy density restricts them to high-power, low-energy applications [25]. Supercapacitors, with higher energy density than capacitors, combine fast charge–discharge capability with long cycle life, making them ideal storage elements [26]. Rechargeable batteries provide the highest energy density but slower charging and discharging rates and limited cycle life, suitable for high-energy and low-power applications [27].

In TENG-based self-powered systems, energy-storage circuits must not only provide high-capacity and efficient storage but also incorporate robust energy-management capabilities. Energy management includes regulating the voltage and current of the TENG output to match the characteristics of the storage element, as well as controlling the charging and discharging processes to ensure safe and reliable operation [21]. In addition, energy management enables power distribution and optimization of the load, thereby improving the overall efficiency of the system [28]. Efficient energy-management strategies are also critical to achieving breakthroughs in Electronic Stability Control System (ESC) performance.

Although TENGs show great potential in the field of self-powered sensors, they still face several technical challenges. High impedance mismatch, pulse energy, and low storage efficiency remain critical bottlenecks that limit their development [29]. TENGs usually exhibit high internal resistance, while most electronics or sensors have low input impedance, and this impedance mismatch can lead to significant energy loss and reduce the overall efficiency of the system [30]. To overcome this problem, it is often necessary to design suitable impedance matching circuits to match the output impedance of TENGs with the input impedance of the load, thus realizing efficient energy transfer. The electrical energy output from TENGs usually has a pulsed characteristic, generating high voltage or current for short periods while remaining close to zero for most of the time, and this pulsed energy is stored less efficiently, which leads to a waste of energy [21]. Special energy-storage circuits are therefore required to improve pulse-energy storage efficiency, which can quickly capture the pulse energy generated by the TENG and store it efficiently [31]. In addition, the output characteristics of the TENG are affected by the mechanical excitation frequency, amplitude, and environmental factors [32], introducing uncertainties that complicate energy-storage design. Adaptive control strategies are used to improve the robustness and adaptability of the energy storage circuit, and the parameters are dynamically adjusted according to the actual output characteristics of the TENG to realize the optimal energy storage effect [33]. With the development of AI, machine learning now offers new opportunities for predicting TENG behavior and optimizing storage-circuit parameters [34]. In addition, the performance of TENG is significantly affected by ambient humidity and temperature. Elevated humidity leads to the adsorption of moisture on the surface of TENG, which reduces the generation and accumulation of friction charge, and elevated temperature affects the performance of TENG materials and reduces their energy conversion efficiency [35]. As a result, materials engineering can fundamentally enhance environmental robustness, while alternating hydrophilic and hydrophobic microzone structures inhibit water film formation [36]. Self-healing PDMS guarantees output stability over a wide temperature range [37]. TENGs have many advantages as a self-powered sensor for monitoring mechanical signals without any external power supply, since the electrical output generated by the mechanical triggers directly serves as a monitoring signal [38].

However, only a few review studies have focused on TENGs in the context of energy storage and power management. In this paper, we systematically summarize key advances in TENG-based energy storage, power management, and representative applications; analyze the technological bottlenecks and outline future research directions; and provide theoretical guidance for the engineering implementation.

## 2. Basic Contradiction Between TENG Output Characteristics

### 2.1. High-Voltage/Low-Current Characteristics

TENGs typically exhibit high-voltage and low-current output characteristics, as summarized in Table 1. Contact-separation (CS) TENGs generally deliver open-circuit voltages of 0.1–15 kV and short-circuit currents of 1–100 μA [39]. Free-rotating disk (FRD) TENGs, due to their continuous operation, can produce currents of 0.1–2 mA, although the voltage decreases to 0.5–5 kV. Under low-frequency mechanical excitation, TENGs can reach power densities up to 500 W/m^2^, far exceeding electromagnetic generators; however, because of their pulsed high-voltage characteristics, the actual utilization efficiency in circuits is often below 10% [40]. By incorporating electrostatic vibration switches (EVSs), the output impedance can be reduced to below 0.001 Ω, alleviating the mismatch problem between TENGs and external circuits [38].

**Figure 2:**
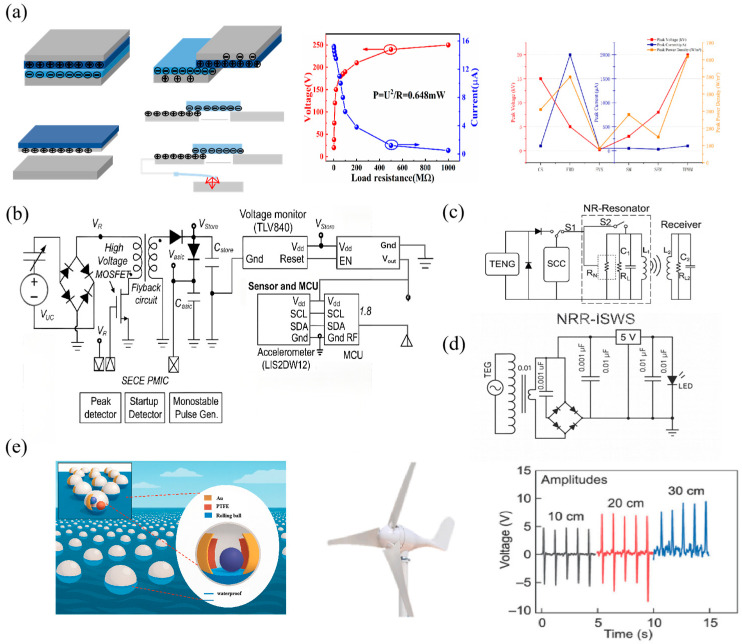
Output characteristics of TENGs and corresponding impedance-matched storage circuits. (**a**) Structural typologies of TENGs and their corresponding high-voltage–low-current profiles. (**b**) A miniaturized wireless communication module powered by an SECE energy management circuit. (**c**) NRR-ISWS system and conventional LC-ISWS FFT spectra comparison. (**d**) Demonstration of a TENG-powered integrated charging system for small electronics using impedance-transformed circuits. (**e**) Mechanical excitation irregularities that affect TENG output stability. Reproduced with permission from ref. [46]. Copyright 2018, Elsevier. Reproduced with permission from ref. [47]. Copyright 2018, Elsevier. Reproduced with permission from ref. [48]. Copyright 2018, Elsevier.

This output behavior is markedly different from the low-voltage, high-current characteristics required by conventional electronics, creating challenges for energy-storage circuit design. As shown in Figure 2a, the high voltage arises from the large surface charge density of the friction material, originating from contact charging caused by electron-cloud overlap. The low current is determined by the high internal resistance of the TENG, consistent with the charge-source model [41]. Although VOC can reach the kilovolt level, the practical operating voltage typically drops to 10–100 V under load conditions due to impedance mismatch, resulting in energy-transfer efficiencies below 30%.

High-voltage outputs demand storage circuits with strong voltage-withstand capability to prevent device breakdown. kV-level pulses may damage conventional semiconductor switches, requiring the use of gas switches or diode chains; meanwhile, μA-level currents may take up to 2 h to charge a 1 F supercapacitor to 3 V, constraining real-time energy supply [42]. Voltage-doubling and rectification circuits can be employed for voltage adaptation, but they incur 30–50% energy losses [43].

Furthermore, the high internal resistance of TENGs limits current output to the μA–mA range, while the operating voltage can plummet to 10–20% of Voc under load due to impedance mismatch. This low-current output necessitates storage circuits with high sensitivity and ultra-low power consumption to maximize usable energy [44]. At the same time, the high-voltage/low-current profile imposes strict requirements on the choice of storage components: traditional capacitors may fail to withstand kilovolt-level inputs, and although supercapacitors offer higher energy density, their relatively high internal resistance can further restrict current delivery [45].

The output voltage and current of TENGs are also affected by the friction material, structural design, mechanical excitation, and other factors, as shown in Table 1. Different friction materials exhibit distinct surface charge densities and friction coefficients, which directly affect TENG output [49]. The structural design also plays a decisive role: the work-function difference between materials determines the achievable charge density. When the difference exceeds 1 eV, the surface charge density can reach 150–250 μC/m^2^. Moreover, when the contact frequency is above 10 Hz, the output power increases linearly with frequency, though higher frequencies accelerate material wear [50]. In addition, parameters of the mechanical, such as the frequency, amplitude, and waveform, directly govern the energy-generation rate and output power of the TENGs. Therefore, these factors must be comprehensively considered when designing energy-storage circuits in order to achieve optimal energy-storage performance [51].

**Table 1:** Output characteristics of TENG in different modes.

Working Modes	Voltage Range	Current Range	Peak Power Density	Applicable Scenarios
CS [43]	1–15 kV	1–100 μA	310 W/m^2^	Vibration Energy Harvesting
FRD [44]	0.5–5 kV	0.1–2 mA	500 W/m^2^	Wind/water harvesting
EVS [45]	50–200 V	10–50 μA	83.6 W/m^2^	Micropower Management Circuits
Sliding Mode [52]	0.1–3 kV	5–50 μA	280 W/m^2^	Linear motion energy harvesting
Single-Electrode Mode [53]	0.5–8 kV	0.5–30 μA	150 W/m^2^	Wearable device
Triboelectric- Piezoelectric Hybrid Mode [54]	2–20 kV	10–100 μA	620 W/m^2^	High frequency mechanical vibration

### 2.2. Impedance Mismatch Loss

Enhancing the energy-transfer efficiency of TENGs critically depends on resolving intrinsic impedance mismatch, which gives rise to two primary energy loss mechanisms. Owing to contact electrification and electrostatic induction, TENGs inherently possess ultra-high internal resistance severely mismatched with the kilo–ohm-level impedance of downstream electronics or storage modules. This mismatch leads to substantial resistive losses, manifesting as Joule heating, and often accounting for a majority of harvested energy dissipation under poor matching conditions, as confirmed by recent studies [55].

In parallel, reactive-power losses arise from the phase conflict between the capacitive output characteristics of TENGs and the inductive behavior of loads, with non-sinusoidal pulsed waveforms further aggravating phase misalignment. These combined mechanisms typically restrict the effective energy delivered to the load to only about 10–30%, thereby significantly constraining the practical performance of TENG-based self-powered systems [56].

To overcome this dilemma, impedance-matching strategies have undergone multidimensional innovation in recent years. For example, in self-powered microsystems, a synchronous electric charge extraction (SECE) power-management circuit has been demonstrated to increase power output by a factor of five compared with a conventional full-bridge rectifier [57], as illustrated in Figure 2b. In another approach, negative-resistance LC resonators have been integrated into TENG-based wireless sensing platforms, yielding experimentally verified improvements in accuracy and signal stability [46], as shown in Figure 2c. Transformer-based matching strategies have also been investigated, where the equivalent impedance is regulated through the turns ratio. For instance, Lee et al. (2024) employed a miniature high-frequency transformer to enhance the impedance matching of TENGs with low-impedance loads by fivefold while reducing Joule heat losses by 40% [58], as shown in Figure 2d.

Collectively, these methods optimize the relationship between TENG output impedance and load input impedance through static matching, dynamic regulation, and cross-device synergy. Such diversified approaches provide multiple technological pathways to maximize power-transfer efficiency and unlock the potential of TENGs in micro-energy harvesting and self-powered sensing applications.

### 2.3. Mechanical Incentive Irregularity

The output characteristics of TENGs are closely correlated with the frequency, amplitude, and waveform of mechanical excitation [59]. In practical scenarios such as human movement, wind, and water flow, excitation is often irregular, which seriously compromises the stability of TENG output, as shown in Figure 2e [60]. For example, wind-speed fluctuations can induce output-power variations of up to ±40% in rotary TENGs [61]. Meanwhile, irregular body motion can reduce the energy-capture efficiency of contact-separation TENGs by more than 50% [62]. Such variability poses a major challenge to the consistency of energy-storage systems.

To address this problem, robust energy-storage circuits must be designed to accommodate irregular excitation and ensure stable output. A common approach is to incorporate buffering elements—such as capacitors or supercapacitors—to absorb TENG-output fluctuations and smooth voltage/current delivery [63]. Component selection and parameter optimization depend on application-specific conditions. For instance, when excitation frequency exceeds 5 Hz, double-layer capacitors with response times shorter than 10 ms are recommended. For high-magnitude excitation with a coefficient of variation greater than 0.3, hybrid supercapacitors with capacities above 1 F should be employed [64]. Formula (1) describes the calculation of the capacitance required to buffer the pulsed TENG output under irregular mechanical excitation [65].

(1) $$C=\frac{2E_{max}}{∆V^{2}}$$

which $E_{max}$ is the maximum pulse energy and ∆V is the allowable voltage fluctuation. 

Adaptive control strategies are also effective in maximizing energy storage by monitoring mechanical excitation in real time and dynamically adjusting the parameters of energy-storage circuits [66]. Artificial intelligence further opens new opportunities for adaptive energy management. For example, neural networks can be used to predict human movement patterns and optimize the charging and discharging strategies of storage circuits, significantly improving efficiency and stability. Specifically, an LSTM network can forecast excitation trends within the next 5 s, and by dynamically adjusting the duty cycle of a Buck converter, the energy-capture rate can be increased by 30% [67]. Similarly, combining fuzzy-control algorithms with memristor arrays hardware learning enables dynamic scheduling delays of less than 8.7 ms, while consuming only 12% of the power of a conventional MCU, thus offering an efficient solution for transient applications such as industrial vibration monitoring [68].

In addition, adaptively tuning impedance-matching parameters in response to changes in wind or water flow can effectively enhance energy-harvesting performance [67]. It is worth noting that the energy density of TENGs is a key parameter for ensuring continuous and stable operation, yet output performance remains highly susceptible to external influences such as temperature, humidity, material wear, and fatigue deformation [69]. Therefore, future directions include the development of novel materials, modification of existing materials, and the design of advanced packaging strategies to improve TENG performance. Moreover, the intrinsic high-voltage/low-current characteristics of TENGs limit the capture efficiency of conventional storage components to below 10%, underscoring the urgent need for new storage technologies with high-frequency response and low equivalent series resistance (ESR) [70]. These constraints on impedance, pulse irregularity, and environmental perturbations directly shape the design space of storage elements; accordingly, Section 3 classifies and optimizes TENG-oriented storage technologies to address these challenges.

## 3. Energy Storage Technologies for TENGs

To effectively harness the pulsed, high-voltage, and low-current output of TENGs, energy-storage elements play a pivotal role in buffering, stabilizing, and delivering usable electrical energy. This section focuses on storage devices—including capacitors, batteries, and hybrid systems—with an emphasis on their material configurations, structural innovations, and performance-optimization strategies.

### 3.1. Capacitive Energy Storage

Capacitive energy storage has become one of the key methods for TENG due to its fast charge/discharge and long cycle life. However, the intrinsic bottleneck of low energy density has driven innovations toward high-frequency response and low ESR. Since the pulse width of TENG outputs is typically less than 10 ms, capacitors must capture more than 90% of the energy within a 1 ms response time. Traditional aluminum electrolytic capacitors, with response times up to 100 ms and resulting in about 60% energy loss, have been increasingly replaced by low-ESR ceramic and film capacitors [71].

Buffer-filtering strategies aim to optimize output stability. RC filters, with simple structures and ripple-rejection rates below 40%, are suitable for low-frequency vibration scenarios. LC filters achieve rejection rates above 80%, making them appropriate for medium- and high-frequency applications. Active filters, although having a relatively large volume coefficient of 3.0, offer rejection rates exceeding 95%, which makes them preferable for powering precision sensing [63]. Furthermore, distributed active equalization technology configures independent Buck–Boost circuits for each supercapacitor unit, resulting in a 15% increase in capacity utilization [72].

**Figure 3:**
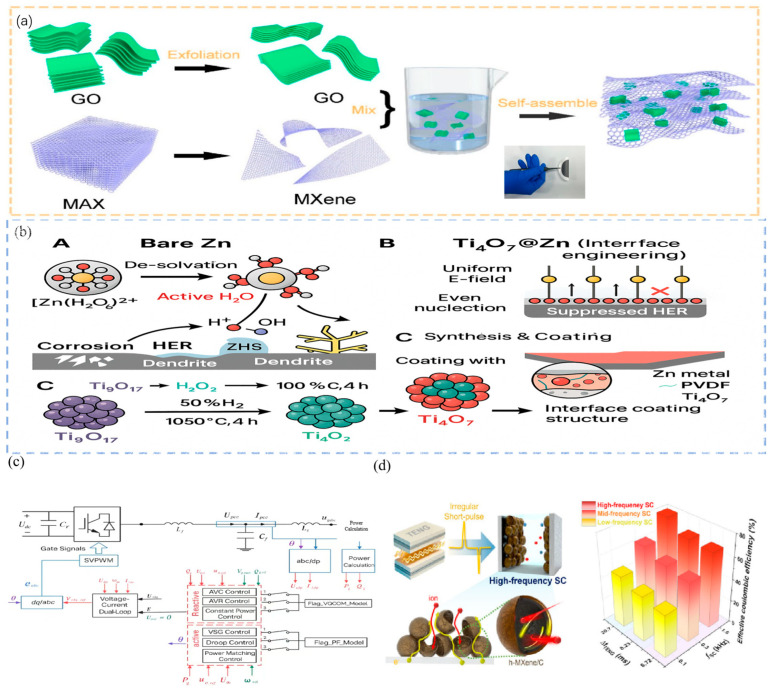
Advancements in energy storage technologies adapted for TENGs. (**a**) Fabrication of a flexible MXene/GO electrode with enhanced ion diffusion for high-rate supercapacitor performance. (**b**) Zn-ion battery electrode design using Ti-based interfacial layers to facilitate rapid charge transfer under TENG pulsed input. (**c**) Grid-type hybrid energy storage system enabling frequency-domain power allocation between battery and supercapacitor. (**d**) Hollow-structured MXene-based electrode with 3D ion transport network designed to match TENG high-frequency pulsed output. Figure from ref. [77], used with permission of the Creative Commons CC-BY.

As an ideal energy-storage component for TENGs, the rate performance of supercapacitors has become a major research focus, with optimization efforts directed at materials, structure, and circuit design. W. Yang et al. reported an MXene/graphene composite electrode that enhances the ion-diffusion rate by fourfold through atomic-layer deposition, achieving 85% capacity retention at a current density of 100 A/g, as shown in Figure 3a [73].

From a materials perspective, N-doped activated carbon improves the electron-tunneling effect via nitrogen atoms, increasing the electrical conductivity by 150% to 120 S/cm. Structurally, the multilevel pore structure of 3D graphene aerogel electrodes shortens ion-diffusion paths, thereby tripling transport rates. At the circuit level, the pulse pre-polarization technique applies a short-duration pulse at twice the rated voltage, reducing ion-diffusion impedance and lowering internal resistance by 30%.

### 3.2. Battery-Based Storage

Rechargeable batteries offer high energy density and relatively long discharge duration, making them attractive for TENG-based self-powered systems that demand a stable and continuous energy supply [74]. In particular, aqueous batteries are particularly favored due to their intrinsic safety and environmental compatibility. However, the relatively slow ion-diffusion kinetics, such as Zn^2+^ transport in aqueous zinc-ion batteries, create challenges when coupled with the microsecond-level pulsed outputs of TENGs, as shown in Figure 3b [75].

To address these limitations, various interfacial-engineering strategies have been proposed. Pre-lithiation and pre-zincation treatments effectively shorten response times to below 100 ms by enhancing ion transfer at the electrode–electrolyte interface [76]. Similarly, introducing Ti-based interfacial layers or surface coatings improves charge-transfer kinetics and prolongs cycle life. Collectively, these material-level innovations enable rechargeable batteries to better accommodate pulsed charging while ensuring stable long-term operation.

Beyond electrode modifications, research has also emphasized material composition and electrolyte design. For example, high-concentration electrolytes and functional polymer additives have been shown to suppress dendrite growth and enhance cycling stability. Advanced cathode materials, such as MXene composites or layered oxides, can further increase energy density while preserving fast ion transport [78]. These advances underscore the potential of rechargeable batteries as complementary storage elements to supercapacitors in hybrid energy-storage systems, where they provide long-term, high-capacity energy buffering.

In summary, rechargeable batteries are essential components of TENG-based self-powered systems, offering high-energy density and long-term operational stability. Although they are less suitable than capacitors for directly capturing high-frequency pulses, recent advances in material engineering and interfacial design have markedly improved their responsiveness.

### 3.3. Hybrid Storage Energy Systems

The capacitor–battery hybrid storage system has emerged as a core strategy for optimizing TENG energy utilization by synergistically combining the high-power density of supercapacitors with the high-energy density of secondary batteries. This configuration not only addresses the mismatch between the TENG pulsed outputs and the frequency-response characteristics of storage devices but also significantly enhances the dynamic response capability and cycle life of the overall system. The topology of hybrid storage systems has evolved from simple parallel mode to hierarchical management schemes based on frequency-domain response. For example, Liu et al. proposed a “heterogeneous voltage-source” parallel structure in a grid-type hybrid energy-storage system, where the Li-ion battery regulates low-frequency, high-capacity power, while the supercapacitor compensates for high-frequency, small-amplitude fluctuations. As a result, the system response delay was reduced to the millisecond level, and the decay rate was lowered by 40% over the life cycle, as shown in Figure 3c [79].

At the circuit level, pulse-charging strategies establish current-distribution pathways by introducing milliohm-level resistors to synchronize and differentiate the charging between the Li-ion battery branch and the supercapacitor branch. Experimental results demonstrate that this approach increases charging speed by 1.8-fold compared with traditional methods and reduces the polarization loss by 32%.

The high-frequency response of supercapacitors is critical for efficiently storing TENG pulse energy. In 2024, MXene-based hollow-structure electrodes were developed with a three-dimensional ion-channel design, raising the characteristic frequency to above 10 kHz and achieving charging efficiencies up to twice those of conventional carbon-based capacitors under TENG pulse input, as shown in Figure 3d and Table 2 [77]. These materials are well adapted to the dynamic range of TENG pulse widths from milliseconds to seconds, achieving an energy-capture rate exceeding 85%. In addition, hierarchical porous carbon electrodes accelerate ion diffusion through integrated microporous, mesoporous, and macroporous channels, maintaining 95% capacity retention at a current density of 1 A/g. This structure makes them particularly suitable for rapid charging and discharging under the irregular mechanical excitation of TENGs.

**Table 2:** Summary of recent energy storage strategies tailored for triboelectric nanogenerators.

Method	Storage Type and Material	Frequency and Pulse Response	Charging Efficiency and Output	Result Summary
Hybrid TENG-SC with MXene Electrodes [80]	Supercapacitor with hollow MXene structure	High-frequency compatible with TENG pulses	Charging efficiency doubled; improved power density	Fast response, good matching but electrode cost. Suitable for wearable and short-pulse environment charging. Fast charging systems.
Flexible Self-Charging SCs [80]	Wearable supercapacitors	<1 ms response; suits high-frequency TENG	30–70%	High flexibility but poor deployment stability and temperature and humidity sensitivity. Suitable for flexible devices and portable sensing.
Hybrid Battery-SC Energy Units [81]	Integrated SC and lithium battery	Handles low-frequency steady-state; high-frequency bursts	Stable delivery, energy-power balanced	Strong continuous power supply capability and stable efficiency but bulky and complex; For edge IoT, uninterruptible power systems.

Table 3 compares typical performance metrics of different storage technologies, including energy/power density, cycle lifetime, pulse response, efficiency, and self-discharge. It further emphasizes their compatibility with TENG output characteristics while outlining key limitations that must be considered for practical implementation. Nevertheless, achieving high end-to-end efficiency and a regulated power supply requires power conditioning capable of extracting charge near the extrema of each pulse, performing wide-range impedance transformation, and stabilizing the output for diverse loads. These stringent requirements have motivated the development of PMC schemes, encompassing switch-device selection, energy-extraction topologies, and regulation strategies co-designed with the chosen storage element.

**Table 3:** Comparative summary of TENG energy storage component applications.

Technology	Energy/Power Density	Cycle Life/Pulse Response	Efficiency/ESR	Self-Discharge	Compatibility with TENG	Disadvantages
Dielectric capacitors [82]	0.1~1 J·cm^−3^/extremely high power density	>10^7^ cycles/μs-level pulse capture	>95%/very low ESR	Very low	Excellent: as first-stage buffer for high-voltage pulses	Very low energy density; volume limits
Supercapa- citors [83]	2~8 Wh·kg^−1^/1–10 kW·kg^−1^	104~105+ cycles/ms-level response	90~95%/low ESR	Significant	High: suitable as second-stage cache and stabilizer	High self-discharge; balancing and over-voltage issues
Lithium-ion batteries [84]	150~270 Wh·kg^−1^/moderate power	500~2000 cycles/slow pulse acceptance	~90%/moderate ESR	Low	Medium: long-term energy buffer with front-end management	Poor direct match with high-impedance pulse sources; needs buffer/SECE
Hybrid Supercapa- citors [85]	20~80 Wh·kg^−1^/1~5 kW·kg^−1^	103~104 cycles/1 ms~100 ms response	High	Moderate	Medium–High: balances energy and power; suitable for wearables	Materials cost; self-discharge in some systems
Battery–supercapa citor hybrids [86]	Tunable by ratio and EMS/peak power from capacitor	Improved lifetime/ frequency-domain synergy	Depends on EMS	Governed by capacitor side	High: optimal for TENG	Higher system complexity, cost, and volume

## 4. Power Management Circuit (PMC) Design

**Figure 4:**
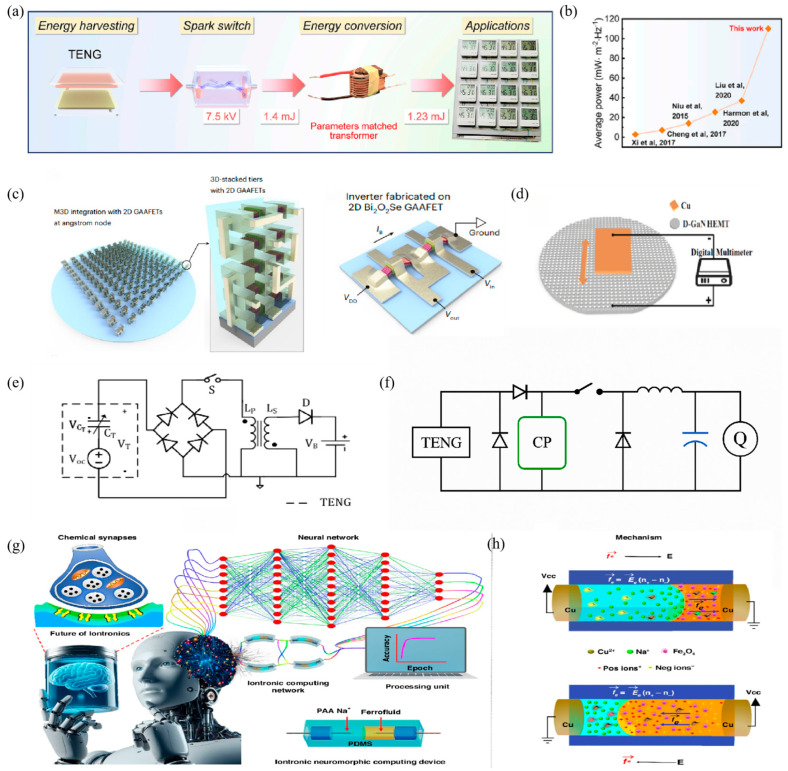
Advanced PMC designs tailored for TENGs. (**a**) TENG output triggering an automatic spark switch for pulse discharge. (**b**) Comparative chart of average output power of various TENG-PMC systems. (**c**) Diagram of 2D Bi_2_O_2_Se/Bi_2_SeO_5_ GAAFET-based logic circuit for ultra-low-power TENG sensing systems. (**d**) SEM image and system integration of Cu/etched D-GaN HEMT architecture tailored for high-efficiency TENG energy extraction. (**e**) SCE circuit with fixed timing switch mechanism. (**f**) Buck-regulated topology added to improve output voltage stability and match load requirements. (**g**,**h**) Iontronic fluidic memristor and ionic liquid channel structure demonstrating neuromorphic adaptation under TENG-powered input. Reproduced with permission from ref. [88]. Copyright 2021, Elsevier. Reproduced with permission from ref. [89]. Copyright 2025, Springer Nature. Reproduced with permission from ref. [90]. Copyright 2024, Elsevier. Reproduced with permission from ref. [91]. Copyright 2024, Elsevier. Reproduced with permission from ref. [92]. Copyright 2023, Elsevier. Reproduced with permission from ref. [93], used with permission of the Creative Commons CC-BY.

In a TENG self-powered system, the performance of PMCs directly dictates the energy-conversion efficiency and system reliability. As the core components of PMCs, the selection and control strategy of switching devices are particularly crucial. Traditional mechanical switches are suited to the high-frequency pulsed outputs of TENGs due to their slow response speed and limited lifetimes. Consequently, advanced technologies such as gas switches and solid-state switches have been rapidly developed, as shown in Figure 4 and Table 4.

In addition to conventional SCE and buck-regulated topologies, recent breakthroughs have demonstrated the remarkable potential of customized PMCs for TENGs. Wu et al. reported that by precisely tuning capacitance and breakdown potential, a TENG–PMC system could deliver pulsed currents of up to 9.8 A with a peak power of 325 kW, and achieve maximum instantaneous currents of 81.2 A. In long-duration operation mode, the circuit maintained a constant output voltage of 1.7 kV with a crest factor of 1.005, enabling 464 LEDs to be continuously powered for 13 min after only 2.5 min of energy harvesting [87].

**Table 4:** Comparison of different switches.

Classification	Switch Type	Pressure Range	Response Time	Conduction Loss	TENG Adaptation Scenarios
Passive Switches	Diodes [94]	<100 V	10–100 ns	0.7 V	Low-voltage and rectification
	GDTs [95]	0.1–20 kV	0.1–1 ms	0.1 V	High-voltage pulse capture
	MEMS plasma [96]	0.3–5 kV	13.2 ns	0.05 V	High-frequency LC resonant circuit
	Spark switch [90]	0.1–20 kV	3–7 ns	0.1–0.12 V	High-voltage pulse capture, industrial environmental monitoring
Active Switches	Memristors [89]	±1–3 V	<0.1 ns	<1 fJ/bit	Brain-like energy management, adaptive control
	GaN HEMT [92]	100–650 V	2–5 ns	<0.1 Ω	High-frequency Buck conversion
	2D GAAFETs [91]	<100 V	<0.1 ns	0.05 V	High-frequency LC resonance, sense and memory integration
Trigger Switch	Mechanical synchronous [96]	<1 kV	µs–ms (depending on trigger)	High efficiency at peak conduction	Pulse energy extraction, proof-of-concept demonstrations
	Electrostatic Vibration Switch [97]	<500 V	ms-level	Ultra-low static loss	Micro-energy harvesting, low-power sensing
	Gas Discharge Trigger Switch [98]	0.1–20 kV	3–7 ns	Low conduction loss	High-voltage pulse capture, industrial applications

Practical demonstrations further highlighted the applicability of this design: a self-powered cathodic-protection system operated for 8 h following just 2.5 min of charging, while a pest-control experiment achieved nearly 100% mortality. These advances not only enhance the stability and scalability of TENG-based energy systems but also offer practical solutions to the long-standing challenges of low-current output and unstable performance under irregular mechanical excitations.

### 4.1. Switch Device Selection

#### 4.1.1. Passive Switches

Passive switches—such as diodes, gas-discharge tubes (GDTs), MEMS plasma switches, and spark switches—operate without external power supplies, making them suitable for cost-sensitive applications or scenarios that do not require complex control. Diodes utilize unidirectional conduction for rectification, but their ~0.7 V forward-voltage drop introduces non-negligible energy losses [99]; GDTs rely on gas-ionization mechanisms to withstand voltages in the range of 0.1–20 kV, demonstrating excellent capability for capturing kV-level pulses from TENGs [88]. MEMS plasma switches achieve nanosecond-level response through microcavity discharges without external bias, making them particularly effective for high-frequency LC resonant circuits. Spark switches, triggered by breakdown under high-voltage pulses, feature response times of 3–7 ns and extremely low conduction loss, making them highly effective for high-voltage pulse capture and industrial environmental monitoring, as illustrated in Figure 4a,b. Experimental results have demonstrated that with this type of energy-management circuit, a 0.01 m^2^ TENG can deliver a pulsed power density of 11.13 kW m^−2^, setting a new benchmark for energy-management performance [89].

#### 4.1.2. Active Switches

Active switches, represented by MOSFETs and memristors, enable efficient energy management through dynamic control. MOSFETs are widely employed in high-frequency scenarios due to their low on-resistance and microsecond-level switching speeds. However, they can consume more than 70% of the static power at μA-level inputs, necessitating the use of zero-static-power LC resonant drive technology to mitigate losses [95]. As a new type of resistive device, memristors offer unique advantages for TENG-based systems. By applying a programmed pulse with a 1 V amplitude and a 10 ns pulse width, memristors can reduce ion-migration losses by up to 90%, while simultaneously enabling complex logic control and optimal energy distribution [98].

#### 4.1.3. New Switching Technology

In recent years, two-dimensional gate-all-around field-effect transistors (2D GAAFETs) and gallium nitride high-electron-mobility transistors (GaN HEMTs) have emerged as prominent research directions for TENG power-management applications. Tang, J. et al. developed a 2D GAAFET that achieved an on-state current of 1 mA/μm at a low voltage of 0.5 V along with a switching ratio of 10^8^ at <1 V and an intrinsic delay of only 1.9 ps, making it particularly attractive for power-sensitive TENG sensor nodes, as shown in Figure 4c [90]. Luo et al. enhanced the performance of depletion-mode GaN HEMTs (D-GaN HEMTs) through inductively coupled plasma surface patterning. By carefully controlling etching depth and pattern density, an ordered diamond-like surface structure was formed, thereby increasing surface roughness and charge density, as illustrated in Figure 4d [91].

In addition to conventional passive and active switches, several types of triggering switches have recently been proposed to mitigate the high internal impedance of TENGs and improve charge-transfer efficiency. Synchronously triggered mechanical switches operate by closing at the voltage peak of the TENG output, thereby significantly enhancing the energy extracted per pulse, although their long-term durability and integrability are constrained by the use of mechanical components [96]. Electrostatic vibration switches, on the other hand, rely on variations in electrostatic fields induced by ambient vibrations to achieve self-triggered conduction. These devices feature ultra-low power consumption and adaptive operation, making them suitable for micro-energy harvesting and low-power sensing systems [97]. Gas-discharge switches utilize high electric fields to induce gas ionization, enabling nanosecond-scale breakdown conduction. They are highly effective for capturing high-voltage pulses and reducing transient equivalent impedance; however, their relatively large size and higher energy consumption restrict their use to high-voltage or specialized industrial applications [98]. The development of these triggering switches highlights a shift in TENG power management from static impedance matching toward dynamic triggering control. By complementing synchronous charge extraction and other advanced circuit topologies, they enrich the overall strategy space for achieving efficient TENG energy management.

### 4.2. Energy Extraction Topology

Efficient energy extraction is essential for overcoming the impedance mismatch and pulsed nature of TENG outputs. Beyond conventional rectifiers, switched-capacitor and charge-pump circuits have been widely investigated. By dynamically reconfiguring multi-capacitor–switch networks, switched-capacitor circuits can capture pulsed outputs on sub-millisecond timescales, reducing equalization losses in supercapacitor banks by nearly 50%. Charge-pump circuits additionally provide voltage step-up for low-amplitude TENG pulses, thereby enhancing compatibility with storage capacitors and flexible electronic devices [100]. Although these approaches may incur switching losses of 30–40%, their structural simplicity and scalability make them particularly attractive for cost-sensitive and resource-constrained applications.

Among advanced rectification schemes, synchronized charge extraction (SCE) circuits maximize charge transfer by switching at the voltage peak, thereby boosting pulse-energy capture efficiency from ~18% to over 40% [92], as shown in Figure 4e. Resonant SCE (r-SCE) further reduces switching losses by ~40% through LC resonance, while hybrid SEF–SCE topologies increase extraction efficiency to ~68% by employing bias-flipping techniques [101]. Collectively, these innovations substantially enhance energy-conversion efficiency while preserving compact circuit footprints.

Voltage adaptation represents another critical challenge, as TENGs typically produce kilovolt-level outputs, whereas downstream electronics generally require low-voltage operation. Buck converters are commonly used to step down high TENG voltages into the 3–5 V range suitable for microelectronics, with optimized charge-pump–buck hybrids achieving average output powers exceeding 100 μW. Beyond buck regulation, boost topologies are also essential for interfacing with battery-based storage. Inductive boost converters enable efficient high-current charging of large-capacity batteries, while capacitive boost circuits are preferred in wearable or miniaturized systems due to their structural simplicity and compact form factor [4]. Collectively, these rectification and regulation topologies provide a comprehensive toolkit for stabilizing and delivering TENG energy efficiently to diverse storage modules. Xiao et al. proposed a TENG energy-management strategy that integrates a charge-pump circuit with a buck converter and systematically investigated their working mechanisms as well as the optimization of circuit parameters. The coupled design enabled the TENG device to exhibit excellent output performance, achieving an average power of 100.7 μW/Hz and a voltage gain of 4.02 V, as shown in Figure 4f [93].

### 4.3. Adaptive and Intelligent Circuits

The output characteristics of TENGs are strongly influenced by the amplitude, frequency and waveform randomness of the mechanical excitation. To enhance system robustness, adaptive circuits maintain efficient energy capture by dynamically adjusting impedance and topology parameters. The technical evolution of such circuits can be broadly categorized into mechanical–electrical feedback regulation, magnetically assisted non-contact regulation, and amnesia–AI synergistic control. Excitation frequency is detected by piezoelectric sensors, and the capacitance of the LC resonant cavity is tuned accordingly to achieve matching for optimal energy extraction. In addition, magnetically assisted regulation leverages the levitation-based stator–rotor pitch adjusts dynamically in response to wave impact forces, thereby stabilizing output amplitude. For example, magnetically assisted adaptive circuits have demonstrated output fluctuation of less than 5% under sudden wind-speed variations [102]. Khan et al. proposed integrating an ionic fluidic memristor (IFM) with low input impedance into a ferromagnetic fluid (FF)-based TENG. This method enables FF-TENG to incorporate contact-separated electromagnetic signals while reducing input impedance, thereby improving energy harvesting performance. Moreover, the harvested energy can autonomously power the ionic fluidic memristor, supporting self-powered computing functionalities [94].

#### 4.3.1. Mechanical-Electrical Feedback to Regulate Variable Impedance

Using piezoelectric materials, MEMS systems, and related technologies, the mechanical deformation or vibration of a TENG can be converted into an electrical signal, which serves as feedback to regulate variable circuit components such as resistance, capacitance, or inductance [103]. This dynamic adjustment enables real-time impedance matching and improves energy-transfer efficiency. The method offers fast response speed and high precision; however, the circuit complexity and high cost limit its applicability to scenarios with stringent performance requirements.

#### 4.3.2. Machine Learning and Hardware Co-Optimization

**Figure 5:**
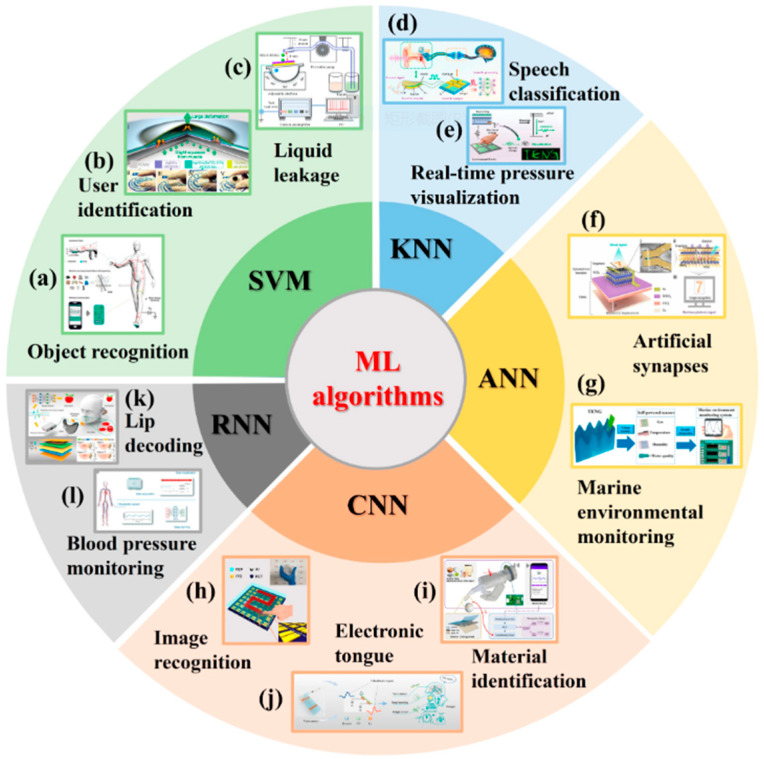
Overview of machine learning integration with TENG-based sensing for adaptive energy management. Figure from ref. [104], used with permission of the Creative Commons CC-BY.

Machine learning algorithms, such as neural networks or support vector machines, are increasingly applied to identify the operating state of the TENG, such as vibration frequency, vibration amplitude, or ambient temperature, and automatically adjust circuit parameters to achieve optimal energy extraction. This approach offers strong adaptability and robustness; however, it requires large training datasets and involves high algorithmic complexity, making it more suitable for environments demanding high adaptability [104]. In practical implementations, multichannel TENG sensors are used for data acquisition to improve stability, but this inevitably increases both the volume and complexity of the data to be processed. Currently, widely adopted machine learning models for flexible electrostrictive TENG elements include Support Vector Machines (SVM), Random Forest (RF), K Nearest Neighbors (KNN), Convolutional Neural Networks (CNN), Recurrent Neural Networks (RNN), Artificial Neural Networks (ANN), as shown in Figure 5.

In summary, the choice of advanced PMC devices and adaptive circuit strategies not only ensures efficient charge extraction and stable power delivery but also lays the foundation for system-level integration.

### 4.4. Energy Storage Efficiency Calculation Methods for TENG

In the study of power management circuits for TENGs, energy-storage efficiency serves as a key evaluation metric. However, the definitions and calculation methods of efficiency reported in the literature are not standardized, often leading to large discrepancies in experimental results under similar conditions. To enhance comparability and scientific rigor, this section systematically summarizes and compares the commonly used efficiency-calculation approaches.

Table 5 presents a classification of these methods, explaining why results may vary significantly across different studies. Such a systematic framework clarifies the underlying sources of discrepancy and provides a more consistent basis for evaluating and comparing the performance of TENG power management circuits.

**Table 5:** Comparison of energy storage efficiency calculation methods for TENG.

Method	Calculation Formula	Applicable Scenarios	Advantages	Limitations/Error Sources
Capacitor voltage method [105]	$E=\frac{1}{2}C(V_{f}^{2}-V_{f}^{2})$	Capacitor/supercapacitor charging	Simple, widely used	Neglects initial energy, self-discharge, and ESR tends to overestimate
Instantaneous power integration [106]	E=∫V(t)i(t)dt	Various circuits and storage ports	Accurate, can separate different losses	Complex measurement requires high-bandwidth sampling
Coulombic efficiency [107]	ηCE=QdisQch	Batteries/electrochemical capacitors	Reflects charge reversibility	Does not directly reflect energy loss
Per-cycle normalization [108]	ηcycle=Estore,cycleEin,cycle	Periodic excitation of TENG	Suitable for comparison across frequencies/amplitudes	Large fluctuation in single cycle requires statistical averaging
Peak/pulse-capture method [106]	Energy ratio per pulse	SECE, triggered switches, pulsed circuits	Highlights peak energy capture	May be misleading if only peak value is reported without average power
End-to-end efficiency [109]	ηCE=EloadEmech	Whole system performance evaluation	Most realistic reflection of actual efficiency	Complex measurement, requires synchronous mechanical–electrical testing

## 5. TENG Energy Storage System Integration Design

### 5.1. Energy Supply–Sensing Co-Optimization

**Figure 6:**
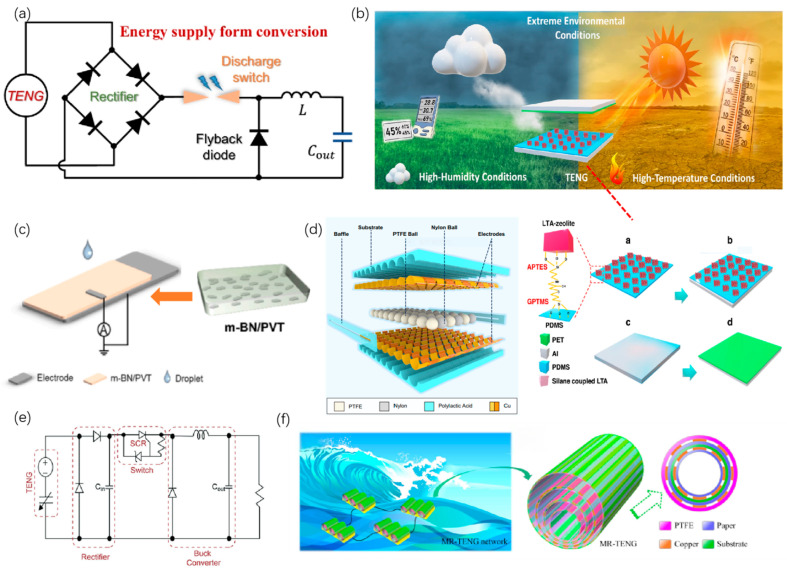
Integrated system-level design strategies for TENG energy storage systems. (**a**) Impedance-matched co-regulation between TENG and switched power circuits for improved stability. (**b**) Moisture-resistant encapsulation for operation in humid environments. (**c**) Schematic of TENG device integrated with temperature-resistant m-BN material. (**d**) Rolling-mode TENG structure designed for rough marine environments with multichannel mechanical coupling. (**e**) Power management topology with switched capacitor chains and multistage SCR triggering. (**f**) Energy harvesting from marine wave motion using an MR-TENG with optimized charge scheduling and series-parallel energy storage management. Figure from ref. [21] with permission of the Creative Commons CC-BY. Figure from ref. [110] with permission of the Creative Commons CC-BY. Reproduced with permission from ref. [115]. Copyright 2023, Elsevier. Figure from ref. [116] with permission of the Creative Commons CC-BY. Figure from ref. [117] with permission of the Creative Commons CC-BY. Figure from ref. [118], used with permission of the Creative Commons CC-BY.

In the integration of TENGs with energy storage and sensing systems, synergistic optimization of energy supply and sensing functions represents a key design strategy for achieving efficient, self-driven, and stable operation. To improve overall system performance, it is essential to synchronously optimize the dynamic matching between the energy-management and sensing-response modules. Li et al. proposed a synergistic mechanism between TENGs and switching-circuit systems, which significantly improved energy conversion efficiency and enabled stable power delivery to the sensing module by matching the TENG output impedance through the system-level switches, as shown in Figure 6a [21] and Table 6. This “waveform-sensing” integrated design substantially enhances the synergy between energy supply and information acquisition. In another approach, Lee et al. developed a high-frequency-response synergistic strategy for a TENG-supercapacitor (SC) hybrid system, demonstrating that optimal matching between SCs and the TENG pulse frequency improves charging efficiency and ensures sufficient dynamic power support during the peak sensing phases, as shown in Figure 6b [110]. More recently, with the rising demand for real-time environmental monitoring, researchers have extended system-level synergistic optimization to include the operating frequency band and response time of sensors alongside the electrochemical characteristics of energy-storage units. This holistic design achieves simultaneous improvement of energy supply and sensing performance [111]. Looking forward, future strategies should emphasize closed-loop system design, beginning with the coordinated regulation of the TENG output spectrum, the response frequency of the energy-storage module, and the bandwidth of sensor-signal acquisition. Such integration is expected to realize the full vision of “self-powered to self-sensing to intelligent feedback” in next-generation autonomous systems.

**Table 6:** Representative machine learning algorithms integrated with TENG-based signal processing systems.

Method	ML Algorithm	Training Data and Features	Accuracy and Real-Time Performance	Result Summary
ML-enhanced Self-powered TENG Sensors [112]	SVM, CNN, RF, LSTM	Signal features: amplitude, frequency, waveform; samples >10 k	Accuracy >90%; latency <100 ms	Robust to complex signals but High training cost; suitable for motion recognition, environmental monitoring, human–computer interaction.
Rotary TENG and NN for RPM detection [113]	Soft-coded NN classifier	RPM range training dataset; 100 s of samples	>90% prediction; 6.6 mW peak; lights 65 LEDs	Real-time stable output but limited to known mechanics; for machine speed monitoring, industrial automation.
GNN-guided Electrode Material Discovery [114]	GNN material prediction model	Doping ratio, structure, energy yield	Output improvement 65–85%, peak 1.12 J/cm^2^	Fast screening, low experiment cost but model dependence; for the direction of material design and performance optimization.

### 5.2. Design for Environmental Adaptation

In TENG-based energy storage systems, environmental adaptation design is a core strategy to enhance the long-term stability and practical usability. Humidity greatly affects the TENG energy output because moisture interferes with contact charge transfer. Researchers have adopted superhydrophobic/superhydrophilic surface treatments and multilayer encapsulation structures, which significantly improve humidity resistance [115] and surface microstructuring, and related strategies have also emerged as key approaches, effectively expanding the applicability of TENGs in high-humidity environments. Temperature fluctuations likewise impair output performance through thermal expansion or conductivity changes in materials. Yuan et al. demonstrated that by introducing temperature-resistant materials, the device maintained stable output under variations from 25 to 100 °C, as shown in Figure 6c. In addition, the rolling-mode TENGs (MO-TENGs) have been structurally optimized to adapt to rough and dynamic environments. Their multilayer channel and rolling-ball designs not only improve power-generation efficiency but also enhance environmental stability via mechanical interlocking structure, as shown in Figure 6d [116]. TENG energy storage systems must integrate material surface engineering, encapsulation design, and dynamic structural optimization to achieve robust resistance against humidity, temperature fluctuations, and mechanical disturbances. Such strategies are essential to ensure autonomous, durable, and long-lasting energy storage applications.

### 5.3. Energy Scheduling and Management

In the integrated design of TENG-based energy storage systems, energy scheduling and management strategies are central to achieving high energy utilization, stable storage, and long-term autonomous operation. Recent research has focused on multiphase electrical energy management circuits (MEH), intelligent switching strategies, and dynamic cooperative control of storage units. Oh et al. developed a multistage power management circuit integrating switched capacitors, rectifier bridges, and boost converters. This system maximized the capture of TENG pulse energy by instantaneously charging C_in_ and triggering SCRs; when connected to a buck module, it charged a 20 µF storage capacitor to 5 V within 1 min, which is nearly three times more efficient, as shown in Figure 6e [117]. Meanwhile, Xia et al. developed a Multi-Roller Structured TENG (MR-TENG), coupling mechanical fluctuations with power management. By optimizing the number of rollers and connection patterns, they significantly improved energy output and implemented a dynamic scheduling strategy for storage devices in series-parallel configuration, as shown in Figure 6f [118]. In addition, Du et al.’s recent review emphasized that multistage energy scheduling strategies are becoming mainstream for addressing the low-frequency characteristics of TENG. These include impedance matching, primary/secondary cache-capacitor switching, dynamic buck modules, and load-based adaptive power allocation, which are crucial for enhancing overall utilization [56]. By precisely controlling timing, dynamically adjusting charge-transfer pathways, and incorporating load-demand self-adaptation mechanisms, it is possible to construct efficient, intelligent, and durable autonomous energy management systems. Such systems hold significant promise for deployment in edge electronics, wearable devices, and IoT applications.

## 6. Application of Cases and Technology Adaptation

### 6.1. TENG Applications in Wearable Devices

**Figure 7:**
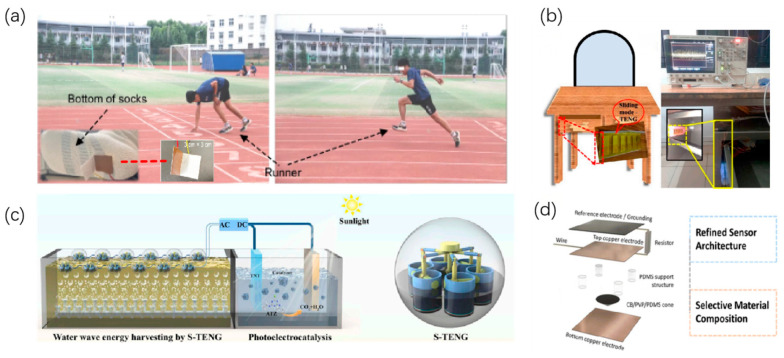
Applications of TENGs in practical self-powered systems. (**a**) Integration of PP-TENG in sports socks for real-time gait energy harvesting and Bluetooth communication. (**b**) TENG-powered self-locking drawer with embedded alarm system for smart security. (**c**) TENG-driven groundwater contamination monitoring system capable of operating wirelessly and autonomously in remote areas. (**d**) Self-powered electronic skin with cone-shaped PDMS sensor array for multidirectional tactile feedback and pressure detection. Figure from ref. [121], used with permission of the Creative Commons CC-BY.

The demand for lightweight, self-driven, and flexible energy units for wearable electronics continues to grow. TENGs have emerged as one of the most important solutions for wearable energy-harvesting systems, owing to their excellent flexibility, lightweight, and high-voltage output, as shown in Table 7. Sheng et al. reported a wearable textile system that seamlessly integrates energy harvesting, storage, and rectification, realizing an on-body self-charging platform for powering portable electronics [119]. Similarly, Deng developed a polyester–paper–cloth composite TENG (PP-TENG) embedded in sports socks, which efficiently harvested human motion energy during daily exercise while simultaneously enabling gait monitoring and data transmission. This device generated an open-circuit voltage of up to 466 V and a peak power output of 930 µW during exercise, highlighting its potential for exercise monitoring and health management, as shown in Figure 7a [120]. Notably, the PP-TENG also exploited its pulsed electrical characteristics to drive a low-power Bluetooth module for wireless data communication, demonstrating that TENGs can provide continuous power support for wearable electronics even in battery-free environments.

**Table 7:** Comparative analysis of system-level TENG integration strategies under environmental constraints.

Method	Integration Strategy	Environmental Adaptivity	Output Parameters	Result Summary
MR-TENG with Rolling Drum Design [121]	Multi-roller and series/parallel scheduling	Saltwater-proof encapsulation	Voc ≈166 V; Isc ≈2.06 μA; 602 μJ in 100 s	High power density, adapted to the marine environment but the installation structure is complex and costly; applicable to blue energy collection, ocean monitoring.
Moisture-resistant PDMS Surface Structuring [117]	3D microstructure and hydrophobic packaging	Stable in humid/rainy environments	Output degradation ≤10% to maintain stable performance	Highly reliable, suitable for wet outdoor environments but encapsulation limits contact crimping; suitable for outdoor wearable, wet area deployment.
TENG-Electrochemical Hybrid for Marine Fuel [116]	Integrated TENG and water splitting	Corrosion- and humidity-resistant design	1910 W/m^3^ volume power; Fuel yield 7.1 mL/min	Integration of energy harvesting, storage and clean fuel extraction but with high system complexity; suitable for ocean energy platforms, ocean sensing and clean fuel production.

### 6.2. TENG Applications in Agricultural and Environmental Monitoring

In the field of environmental monitoring, energy supply remains a major bottleneck restricting long-term deployment. Guo et al. developed a ferroelectric nanocomposite-based TENG integrated with a voltage-multiplier circuit, which tripled the output voltage to over 6 kV and enabled practical high-voltage applications such as sterilization and electronic pest control [122]. Similarly, Qu et al. designed a self-powered groundwater contamination monitoring system that fully utilizes mechanical perturbation energies from environmental sources such as groundwater fluctuation and surface vibrations to achieve long-term monitoring in remote areas without an external power supply. Powered by TENG, the system supports water-quality sensors, signal acquisition, and wireless transmission modules, enabling continuous measurement of groundwater pH, conductivity, and contaminant concentrations, as shown in Figure 7c [123]. These studies not only demonstrate the practicality of TENGs in energy-limited environments but also highlight their potential for broad applications in agricultural, hydrological, and geological monitoring.

### 6.3. Application of TENG in Smart Home and Security Systems

The application of TENGs in smart homes and security has also attracted significant attention. Munirathinam and Chandrasekhar proposed a self-powered door lock and intrusion alarm system based on TENGs. The electrical energy generated during door lock operation or intrusion events is used to trigger both acoustic and visual alarms, thereby realizing security functions without the need for an external power supply, as shown in Figure 7b [124]. In addition, Tang et al. presented the broader role of TENGs in smart buildings, where applications have expanded to include weather-triggered window control, ambient lighting regulation, and disaster alarms. These systems, when integrated with energy management circuits, demonstrate the multifunctional convergence of energy harvesting, sensing, and control [125]. These advances highlight the diverse adaptability and system-level integration capability of TENG technology, underscoring its potential as a cornerstone for the future of smart-home ecosystems.

### 6.4. Application of TENG in Smart Interfaces and Multidirectional Pressure Sensing

Human–computer interaction devices and smart interfaces increasingly demand multifunctional, flexible, and highly sensitive sensors. Complementarily, Guo et al. analyzed the intrinsic energy transfer losses between TENGs and power management circuits and proposed a “trigger-at-open-circuit-peak” strategy, which achieved a record conversion efficiency of 42.5% and established a universal harvesting–storage–regulation framework [21]. Chen designed a self-powered multidirectional pressure sensor based on TENGs. By employing a PDMS/carbon black/pyrrolidone composite structure, the device achieved high-precision pressure sensing over a wide range of 0–70 mmHg while also enabling wireless signal output, as shown in Figure 7d [126]. This TENG-based sensor not only provides a stable self-sustained power supply but also maintains excellent sensing performance under complex mechanical perturbations, thereby demonstrating its strong potential for human–computer interaction, electronic skin, and soft robotics.

## 7. Challenges and Future Developments

Research on energy storage and power management for TENG-based self-powered systems is expected to advance toward higher levels of integration, intelligence, and multifunctionality. With the rapid development of microelectronics and advanced materials, a key trend is the construction of highly integrated power management modules capable of simultaneously achieving impedance matching, waveform rectification, multilevel charge caching, and dynamic voltage regulation within compact packages. Such integration effectively reduces system size, lowers power consumption, and enhances overall conversion efficiency. In parallel, hybrid energy storage systems are receiving growing attention. These hybrid systems not only provide instantaneous high-power output and long-duration storage but also enable bidirectional energy conversion mechanisms to adapt to complex and dynamic operating environments. Moreover, the incorporation of intelligent management technologies is expected to broaden the application boundaries of TENGs. Among the technical bottlenecks, impedance mismatch between the high-impedance TENG source and conventional storage or load devices remains the primary limitation, as it directly constrains energy transfer efficiency. In addition, high-voltage dielectric breakdown in insulating or packaging layers has emerged as another critical obstacle, particularly in high-output or sterilization-oriented applications. Overcoming these challenges is essential for achieving higher efficiency, long-term reliability, and the widespread deployment of TENG-based energy systems.

With respect to applications, low-power IoT platforms—including wearable electronics and distributed sensors—are expected to achieve earlier breakthroughs, given their relatively modest energy demands and tolerance for intermittent power supply. In contrast, biomedical implants represent a highly promising yet more demanding direction. Here, the intrinsic safety and biocompatibility of TENGs offer unique advantages; however, successful clinical translation will require rigorous validation of material stability and the development of standardized encapsulation techniques.

Looking ahead, research should move beyond broad conceptual proposals and instead prioritize AI-assisted adaptive circuits capable of real-time impedance matching under fluctuating conditions, standardized hybrid storage systems that integrate supercapacitors with micro-batteries to provide stable outputs across diverse voltage regimes, and scalable biocompatible materials tailored for wearable and implantable applications. Furthermore, achieving system-level integration—encompassing sensing, communication, and energy management—will be essential to transform TENGs from laboratory prototypes into reliable power platforms for next-generation IoT and biomedical systems.

## Data Availability

No new data were created or analyzed in this study. Data sharing is not applicable to this article.
